# A cure for AIDS: a matter of timing?

**DOI:** 10.1186/1742-4690-10-145

**Published:** 2013-11-22

**Authors:** Iart Luca Shytaj, Andrea Savarino

**Affiliations:** 1 Istituto Superiore di Sanità, Viale Regina Elena, 299, Rome 00161, Italy

**Keywords:** Eradication, Functional cure, Reservoirs, Acute HIV infection, Stem cell transplantation, Vorinostat, Therapeutic vaccine, Auranofin, BSO

## Abstract

Despite the huge clinical success of antiretroviral therapy, several factors such as side effects, requirement of life-long adherence, high cost, incomplete access to therapies and development of drug resistance make the quest for an ultimate cure of HIV/AIDS a worldwide priority of biomedical research. In this respect, several sterilizing or functional cures have been reported in the last years in both non-human primates and humans. This review provides a summary of the main results achieved so far, outlining their strengths as well as their limitations. A synthetic interpretation of these results could be pivotal in order to develop an effective and widely available cure.

## Review

### Introduction

The quest of a cure for AIDS has been defined a “herculean task” [1], given the enormous complexities behind it and the numerous setbacks that have curbed early enthusiasms along the years. The ultimate goal of research for a cure is the complete eradication of the virus from the organism (*i.e.* a “sterilizing cure”), but a more feasible goal may be the achievement of spontaneous drug-free control of the infection without disease progression (*i.e.* a functional cure) [2]. The enormous difficulties that have been encountered in the quest of a cure for AIDS reside in the complex virus/host interplay that is a hallmark of this disease. Infection with HIV is initially characterized by a primary (acute) phase in which the virus is partially controlled by a robust immune response of the host [3]. Unfortunately, this immune response is not sufficient to eradicate the virus from the body, opening the way to the asymptomatic (chronic) phase. The chronic phase is characterized by an initial “steady state” between the virus and the immune system that is then slowly tilted in favor of the former, eventually leading to AIDS in the majority of the patients [4]. Treatment with antiretroviral drugs (ART) can reproducibly decrease viremia to levels below the limit of detection of the routine clinical assays and delays immune deterioration, but is not sufficient to tackle the viral reservoirs or to induce a strong immune response against the virus [5-7]. The viral reservoirs are formed early during acute infection [8] and are exceptionally stable sources of viral persistence [6,9], harboring latent copies of integrated virus that are “invisible” to the immune system and unharmed by ART (5,6,9, for a review on the latency mechanisms, see: [10]). Viral reservoirs can be of both myeloid and lymphoid lineage, allowing a widespread distribution to different compartments such as the central nervous system, the gut-associated lymphoid tissue and the reproductive tract [11]. At a cellular level, central and transitional memory T-cells (T_CM_ and T_TM_) were recently identified as a crucial source of viral persistence during therapy [12]. Additionally, macrophages are regarded as important contributors to this persistence, as well [13].

This review provides an outline of the therapeutic successes in the pathway towards a cure for AIDS. Our description is focused on the results that have so far been obtained in humans or SIV/SHIV infected macaques, which are, among the allowed animal models, those phylogenetically nearest to humans and most closely recapitulating the pathogenesis of human AIDS [14,15]. Recent reports have provided substantial data supporting the view that the path to a cure is a viable research avenue. These new data allow attempting a re-evaluation of the paradigms that have oriented cure-related research and addressing some of the questions that have so far been left unanswered.

### Hit fast, hit hard

Acute infection offers an ideal time window for effective therapeutic interventions [3]. A pioneering demonstration of the therapeutic potential of early treatment was the case report of spontaneous control of viral replication following treatment interruption in the first “Berlin Patient” [16] (not to be confused with Mr. Timothy Brown, the second “Berlin Patient”, see next subchapter). This man was treated during acute infection with a non-standard ART regimen (containing hydroxyurea) and subsequently underwent two structured treatment interruptions (STI). Eventually, after the second STI, the man displayed a long-lasting (19 months, until he was lost to follow-up) spontaneous control of viral load below the assay detection limit (500 copies of viral RNA/mL). Moreover, viral load control was accompanied by immune restoration, with CD4 counts and CD4/CD8 ratio progressively increasing over time [16]. This striking result confirmed those of a previous study by Vila *et al.*, employing a similar drug regimen and achieving as well a long-lasting post-therapy viral load control in two human subjects [17]. However, both studies were uncontrolled, and the two clinical cases described by Vila *et al.* were associated with high CD4 counts and low viral loads before treatment initiation [17]. A fully controlled animal study employing a therapy containing hydroxyurea administered sequentially in the form of multiple ART/STI cycles strengthened these case reports and showed that post-therapy viral load control could be induced in macaques acutely infected with the HIV homolog SIVmac251 [18]. Of note, in all these studies, apart from the early treatment initiation, hydroxyurea may have played a role in the post-therapy viral load control obtained. Hydroxyurea exerts a cytostatic effect by inhibiting the activity of the ribonucleotide reductase enzyme, thus halting the cell cycle at the G1 phase [19]. This effect may hamper viral reservoir maintenance/expansion in T_CM_ and T_TM_ cells that mainly relies on antigen-driven and homeostatic proliferation respectively [12]. Despite these promising results, combinations of hydroxyurea and antiretroviral drugs displayed in some instances high pancreatic and hepatic toxicity [20,21] and consequently hydroxyurea is not recommended for routine treatment of HIV infection, although there is still ongoing research on this topic [22].

Another uncommon ART regimen administered during early infection yielded promising results in a recent study conducted in macaques infected with different SIV/SHIV strains [23]. In some of these animals, a prolonged (more than 8 years) tenofovir monotherapy proved able to induce a spontaneous control of the infection following the final treatment withdrawal [23]. Apart from the early treatment initiation, this result may be due to an effect of tenofovir in selecting suboptimal drug resistance mutations, and the result may also have been contributed by the additional interventions to which the macaques were subjected during follow-up (temporary depletion of CD8^+^ cells and treatment at viral rebound).

Treatment during acute infection has provided some amount of clinical success also with more traditional ART regimens [24-30]. News such as the case report of the cure of an ostensibly HIV^+^ baby treated in the very early phase of the disease [30] and, more importantly, the results of the ANRS VISCONTI study [29] have been hailed with widespread enthusiasm. Of particular note, up to ≈ 15% of the early treated individuals have been shown to display spontaneous control of viremia following STI [27]. However, the rate of post-therapy control following ART administration during the acute phase may be lower (≈5%) according to another report [28]. Moreover, no definite timing and drug composition has been proven to reproducibly induce post-therapy control even in a minority of patients, and several studies have failed to induce any significant reduction in the post-therapy viral set point following treatment during acute infection [31-33].

**Figure 1:**
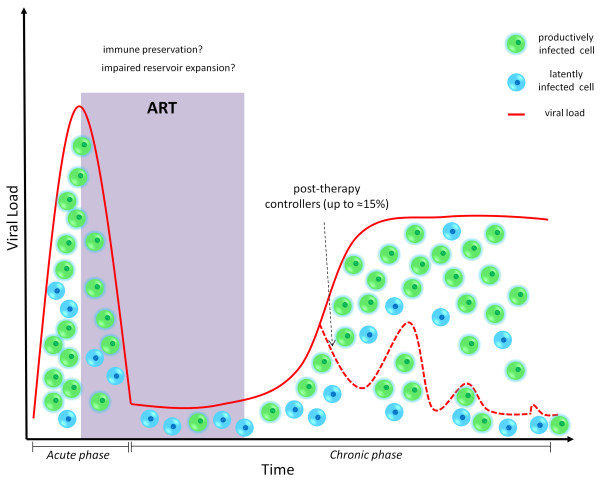
**ART administration during acute infection results in spontaneous control of viral load in a minority of individuals following therapy suspension.** The dotted line depicts representative viral load dynamics of patients controlling the infection following ART withdrawal, as compared to non controllers (solid line). The upper limit of the percentage of controllers is taken from [27]).

Despite these mixed results, the data available indicate that ART administration during acute infection can induce, in a minority of cases, a post-therapy control of the infection which is independent from known favorable genetic backgrounds [29] (Figure 1). Several hypotheses have been postulated to explain the enhanced efficacy of ART treatment during primary infection. The most frequently cited explanations are: 1) the preservation of an efficient immune response [34,35], 2) the induction of a “self-vaccination” after multiple STIs [16,18], and 3) the impairment of viral reservoir formation [27,29,36-38]. A reduced viral reservoir size does not *per se* guarantee successful ART withdrawal [39], but is, even in the most conservative scenario, a promising platform in the quest of a cure. The overwhelming majority of HIV^+^ individuals, however, are diagnosed during the chronic phase of the infection, and a large body of evidence shows that STI protocols (even in the form of short “drug holidays”) are not effective in improving the course of the disease once the chronic phase is established ([40-42], reviewed in: [43]).

### Hit later, hit harder

The mainstream approach to purge the viral reservoirs during chronic infection is a multi-step “shock and kill” therapy [44]. During the “shock” phase, the latent virus harbored in the reservoirs is expected to be pharmacologically reactivated and prompted to resume productive infection. During the”kill” phase, the newly produced virions would be blocked by ART, while the HIV-infected cells are expected to be eliminated by viral cytopathogenicity, or recognized and killed by the immune system. A plethora of compounds have been put forward as candidates to induce the “shock” phase (recently reviewed in: [45,46]). Among these, the most thoroughly investigated are histone deacetylase inhibitors (HDACI’s). Several HDACI’s (*e.g.* valproic acid, vorinostat, panobinostat) have been tested or are currently under investigation in both pre-clinical studies and clinical trials (reviewed in: [47]). Vorinostat [*i.e.* suberoylanilide hydroxamic acid (SAHA)] was recently reported to have a moderate latency disrupting effect in a group of patients previously selected for the responsiveness of their resting CD4^+^ memory T-cells to treatment with this drug *in vitro*[48]. However, preliminary data do not show significant effects of vorinostat on viral reservoir size [49,50], while no data on post-therapy viral dynamics are available so far. Moreover, treatment with combined ART/vorinostat regimens on SIVmac-infected macaques led to mixed or disappointing results [51,52]. More data on the *in-vivo* effects of vorinostat will be available from the two ongoing clinical trials investigating the effects of this drug on individuals under ART (NCT01319383, NCT01365065). For the remaining HDACI’s only data obtained from cell cultures are available at present [53-55], although panobinostat is currently under investigation in a Phase I/II clinical trial (NCT01680094).

Another approach aimed at HIV reactivation from latency involves the use of cytokines (reviewed in [56]). In particular, the use of IL-7 in combination with ART intensification is currently being investigated (NCT01019551). Unfortunately, in two recent clinical trials, the addition of IL-7 to standard ART protocols did not result in viral reactivation from latency [57], and increased the size of the viral reservoir [58], in line with the well-known effects of this cytokine, favoring homeostatic proliferation of T_CM_ and T_TM_ cells [12,58,59].

Despite the enormous efforts that have been put in the study of HIV reactivating HDACI’s and cytokines, the most promising results so far obtained in the quest of a cure for AIDS are not derived from these approaches. The most astonishing result in the field to date, and the first proof of concept for the feasibility of a sterilizing cure during chronic HIV infection, is the case report of the treatment of Mr. Timothy Brown, the aforementioned second “Berlin Patient” [60,61]. Apart from being chronically infected with HIV, this man was diagnosed with acute myeloid leukemia and consequently treated with an aggressive combination of ablative chemotherapy/radiotherapy, immune suppression through drugs and allogeneic stem cell transplantation. Importantly, the donor selected for the transplantation was homozygous for the Δ32 deletion of the *CCR5* gene [60]. This gene encodes for the main coreceptor employed by HIV for entry into cells, and individuals homozygous for the Δ32 deletion (about 1% of the caucasian population) are protected from HIV infection [62]. Following stem cell transplantation, Mr. Brown stopped taking antiretroviral drugs, and has remained off-ART since then, with no signs of disease progression [60,61]. Of note, in spite of an extensive sampling throughout the years, most of the analyses have failed to detect HIV RNA or DNA in blood and tissues, and the HIV-specific antibody titers have steadily decreased over time, thus hinting that a complete eradication may have been achieved [61,63]. Despite the enormous excitement generated by the news of this cure, the scarcity of HLA-DR-compatible *CCR5* Δ32 donors makes it very difficult to replicate the whole experiment. Consequently, several attempts have been made to isolate the contribution of each of the different therapy components. Allogeneic bone marrow transplantation had been employed for treatment of HIV since the first years of the epidemics (reviewed in [64]) and had been even advocated as a possible curing strategy [65]. The most visible difference between these early attempts and the treatment of Timothy Brown is the favorable genetic background of the cells received by the latter, bearing the homozygous *CCR5* Δ32 deletion. Thus, it is not surprising that many investigators have used this observation as a starting point for further studies. In this regard, a gene therapy approach aimed at disrupting the *CCR5* gene (virtually recreating the Δ32 deletion) is currently under investigation in clinical trials (NCT01252641, NCT00842634). In these studies, the disruption of *CCR5* is performed employing zinc finger nucleases in previously isolated autologous cells that are afterwards re-transplanted in the host. The preliminary results released so far do not allow drawing a definite conclusion on post-therapy viral load dynamics, which seem to be quite variable among study subjects, although post-therapy viral load containment may have been achieved in a small subset of individuals that were heterozygous for *CCR5* Δ32 at baseline [66]. Anyway, the zinc finger treated CD4^+^ T-cells have been shown to be able to persist in the organism at least one year after the transplant and have had an enhancing effect on CD4 counts in immunologic non-responders [67].

**Figure 2:**
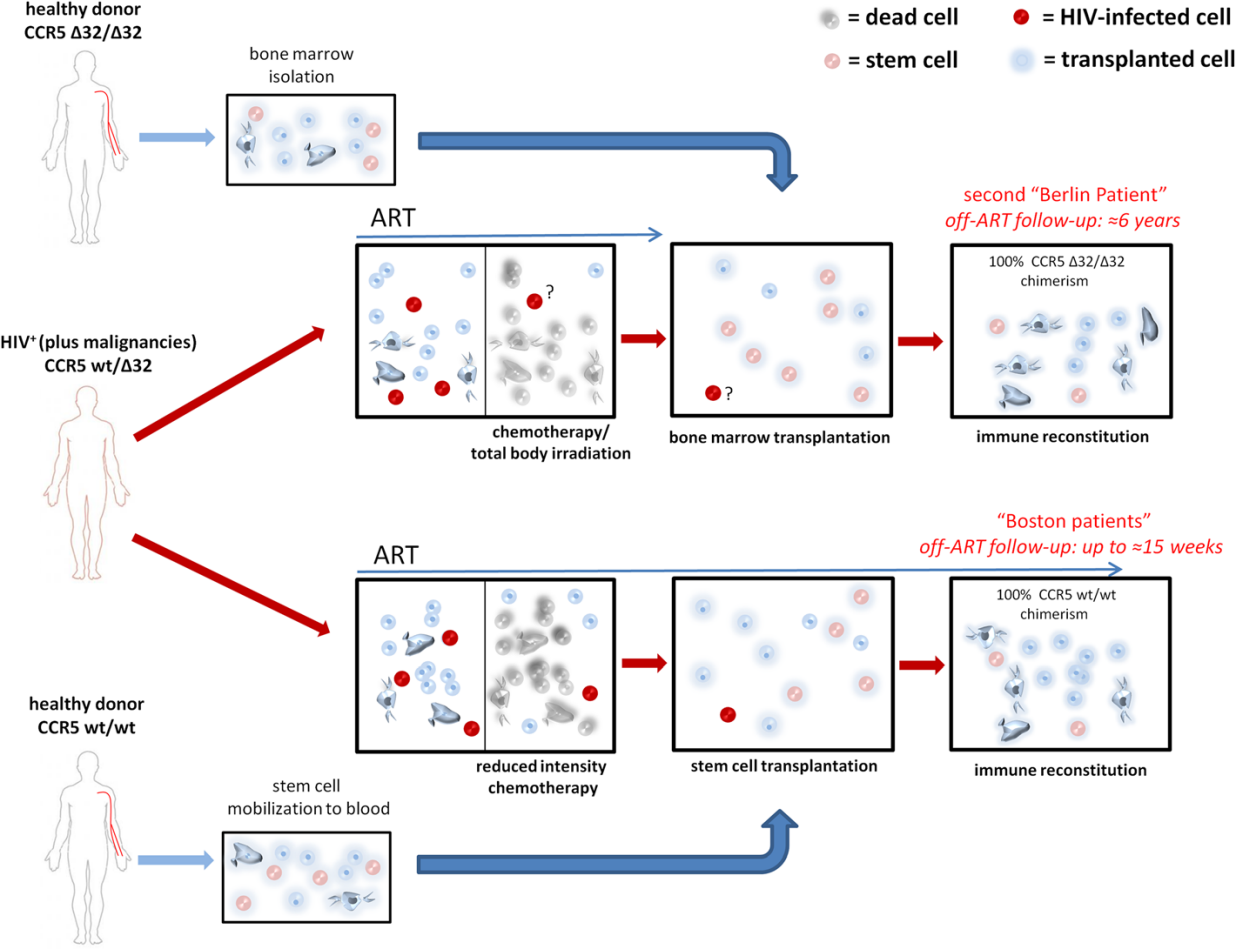
**Schematic representation of the therapeutic interventions received by the second “Berlin patient” and the “Boston patients”.** Although the second “Berlin patient” had received two stem cell transplants, only one is shown for clarity purposes. Note that the length of the arrows indicating the period under ART is meant to provide a qualitative comparison between the ART and transplantation schedules and is not in scale.

On the other hand, recent data indicate that allogeneic stem cell transplantation may possibly lead to a cure also in the absence of the *CCR5* Δ32 mutation. This is suggested by the outcome of the treatment of two HIV^+^ individuals (the “Boston patients”) that had received an allogeneic transplantation of stem cells from *CCR5* wild-type donors. After transplantation, while still receiving ART, these individuals displayed a reduction of viral DNA in peripheral blood to undetectable levels [68]. Further investigation proved that viral DNA could not be detected with large scale analysis in PBMCs and in rectal tissue, and thus STI was attempted in both patients [69]. Following ART interruption, no viral rebound was observed, and, despite the relatively short follow-up, the data available suggest that a cure may have been achieved [69]. An important element of the strategy employed for treating the “Boston patients” may have been the long-term ART maintenance following transplantation which may have blocked viral reseeding before the establishment of a full donor chimerism. This aspect differentiates the “Boston patients” from the second “Berlin patient” in which ART was discontinued from the day of transplantation and viral reseeding was likely hampered by the *CCR5* Δ32 mutation of the transplanted cells. On the other hand, a common feature between these case reports is that the “Berlin patient” and the two “Boston patients” were all heterozygous for *CCR5* Δ32 before transplantation [60,68] (see Figure 2). Although their blood cells were fully replaced afterwards (by homozygous *CCR5* Δ32 cells in the “Berlin patient” and by wild type *CCR5* cells in the “Boston patients”), it cannot so far be excluded that their original *CCR5* Δ32 heterozygous status may have played a role in the clinical outcome. It is known that heterozygosity for *CCR5* Δ32 is associated with slower disease progression [62,70] and the results of a recent study conducted in non-human primates suggest that *CCR5* expression levels may be associated with the size of the viral reservoir [71]. Despite the necessity of conducting further studies on this topic, the high treatment-related mortality of allogeneic transplant [72] hampers the use of this technique as an HIV curing strategy, unless salvage therapies are required due to life-threatening co-morbidities. On the whole, the studies hereto reviewed support the hypothesis that decreasing the viral reservoir size through drugs, coupled to immune system renovation, may be a key to the achievement of a cure.

### Turning back time

**Figure 3:**
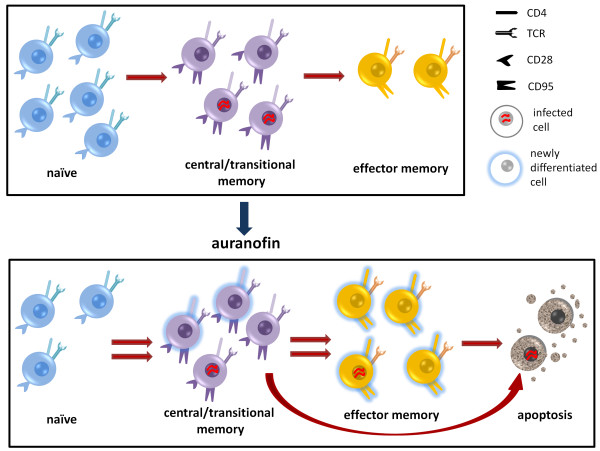
**Treatment with auranofin increases the turnover of CD4**^**+ **^**T-cell subsets and induces a partially selective apoptosis of the memory compartment.** The cell subsets are identified by the expression of the surface markers CD28 and CD95 (naïve: CD28^+^ CD95^-^; central and transitional memory: CD28^+^ CD95^+^; effector memory: CD28^-^ CD95^+^).

From the aforementioned studies, it is evident that the therapies that have resulted in a cure during the chronic phase of the infection bear a much higher degree of risk as compared to the strategies adopted in the acute phase. Thus, the possibility of inducing an acute infection-like scenario in an advanced stage of the disease may represent a unique option to open a new window of opportunity for the therapeutic interventions adopted during the acute phase. A good candidate for this strategy is the gold-based compound auranofin [52] which has been employed for many years in treatment of rheumatoid arthritis [73]. Our group has recently shown the potential of auranofin to act as an anti-reservoir compound *in vivo* when administered to chronically-SIVmac251 infected macaques [52]. Of note, auranofin is able to preferentially induce differentiation/cell death of the memory T-cell compartment including the T_CM_ and T_TM_ CD4^+^ cells which encompass the main viral reservoir [52] (for a schematic representation of the mechanism of action of auranofin, see Figure 3). Beside its anti-reservoir effect, the addition of auranofin to ART was able to prompt a drastic modification of the post-therapy viral load dynamics in chronically SIVmac251-infected macaques [52,74]. Following treatment interruption, the macaques that had received the ART/auranofin combination displayed a sharp viral load rebound reminiscent of an acute infection peak which was in turn accompanied by an increase in specific immune responses in accordance with the typical acute infection scenario [3,74]. From a therapeutic perspective, treatment with ART/auranofin induced a reduction in post-therapy viral load set point (≈1 Log_10_ viral RNA copies/mL of plasma) [52] and, importantly, treatment during the acute infection-like viral rebound showed the potential to mimic the aforementioned effects of early ART administration [74]. Indeed a short cycle of ART during the acute infection-like peak induced a further reduction in post-therapy viral load set-point [74] and, despite being attempted in a low number of macaques (n = 2), would prove effective in the long-term follow-up [74]. The viral load control induced with this therapeutic protocol may have been contributed to by the previous reservoir reduction prompted by auranofin and by the use of an ART regimen containing maraviroc, which, by blocking CCR5, may inhibit antigen-driven proliferation of the viral reservoir [74]. Although the mechanism behind the drastic modification of the viral rebound pattern induced by auranofin remains partially unclear, its effects on the macaque AIDS model suggest that this drug may offer an attractive possibility of successfully applying, to the chronic phase of the infection, strategies that would have been effective only in the early stages.

### Immune enhancement: rejuvenating the immune system?

Enormous efforts have been put in the development of strategies able to boost antibody and/or cell-mediated immune responses against HIV (reviewed in [75]). The curative potential of broad and robust cell-mediated immune responses, in particular by CD8^+^ T-cells, is suggested by the association of such responses with better disease progression resulting in spontaneous drug-free control of viral load in a minority of individuals [76-79]. Thus, drugs able to bolster immunity against HIV-infected cells could represent an ideal tool for prompting, or supporting, a spontaneous control of the infection [75]. A promising compound for enhancing cell-mediated immune responses against HIV may be buthionine sulfoximine (BSO), a glutathione-depleting agent previously tested for cancer treatment in phase I clinical trials [80]. We recently showed that the addition of BSO to the aforementioned ART/auranofin combination is able to promote a significant and long-lasting enhancement of specific immune responses directed against SIVmac Gag [81]. Boosting immunity against Gag is an attractive achievement because several studies have shown that strong anti-Gag immune responses are associated with low viral loads and high CD4 counts both in macaques and humans [82-86]. Moreover, the results of a recent study suggest that CD8^+^ T-cells may reduce the viral reservoir by recognizing Gag antigens produced by latently infected resting CD4^+^ T-cells [87]. In accordance with these studies, enhancement of the immune responses against Gag following suspension of treatment with ART/auranofin/BSO was associated with the obtainment of a functional cure-like condition in a study conducted on a small number of chronically SIVmac251-infected macaques [81].

Partially similar results were obtained using a therapeutic vaccine based on dendritic cells pulsed with whole inactivated virus [88-91]. This vaccine proved able to achieve drug-free control of viral load in a subset of chronically SIVmac251-infected macaques [88] and to induce a reduction of viral load, although moderate, in ART-naïve HIV^+^ subjects [89,90]. Moreover, coupling the vaccine administration to ART induced a reduction in post-therapy viral load set point in some individuals [91]. Of note, the highest viral load reductions observed in ART-naïve subjects were associated with high numbers of Gag-specific CD8^+^ T-cells [89].

A proof-of-concept that strong CD8^+^ (in particular T_EM_)-mediated immune responses can even lead to viral eradication was recently furnished by a preventive vaccine study conducted on macaques challenged with SIVmac239 [92]. Despite all vaccinated macaques becoming infected following multiple challenges with the virus [93], about half of them proved able to spontaneously control the infection and, strikingly, to get rid of the virus completely in the long run [92]. Interestingly, an involvement of T_EM_ cells was also shown, by multiple correlation analysis, in the effects of the auranofin-based therapeutic approach [52].

On the other hand, antibody-mediated immune responses have also proven the ability of inducing post-therapy viral load control [94,95]. In particular, in the recent study of Barouch *et al.*, a cohort of SHIV(env)-infected macaques was treated with wide spectrum neutralizing antibodies [95]. This treatment produced a functional cure in those macaques starting from viral loads of less than 3.5 Log_10_ viral RNA copies/mL of plasma [95]. Of note, this experiment provides an artificial substitution of a “non-functional” immune system with a surrogate functional immunity, *i.e.* the passive antibody transfer. The capability of adoptive antibody transfer to induce a functional cure only in those macaques displaying low baseline viral set points, supports the view that a limited viral reservoir should accompany the immune system renovation.

Finally, also the effects of transplantation strategies on viral load control may be associated with enhancement of immune responses. A study by Villinger *et al.* conducted in chronically SIVmac239-infected macaques showed that adoptive transfer of activated autologous CD4^+^ T-cells may result in spontaneous post-therapy control of the infection [96]. This approach can hardly be employed in humans since it requires cells isolated before the infection, but it suggests that renovation of the immune system is important for obtaining effective immune responses [96]. Of note, autologous stem cell transplantation did not result in a cure of HIV^+^ individuals [97], suggesting that cells isolated following infection may not be apt to prompt immune enhancement. Instead, the likely cures observed following allogeneic transplantation in the “Boston patients” [69] may have been induced or facilitated by the strong immune responses resulting from graft versus host disease, exacerbated by a partial HLA donor/receiver mismatch in one of the two patients, which may have played a critical role for the elimination of the viral reservoirs [68].

## Conclusions

The studies reviewed herein indicate that curing, and even eradicating primate lentiviruses, including HIV-1, could be possible, at least in certain cases (see Table 1). However, it is important to point out that the majority of the “functional” cures that have so far been reported have been obtained during the acute phase or a short time thereafter, *i.e.* at a time in which the viral reservoir and the immune damage are still limited. The design of future therapeutic strategies should address the chronic phase of the disease, affecting the large majority of the HIV^+^ individuals. The most successful approaches tested so far, though still preliminary and/or based on a small number of cases, strongly suggest that the path to a cure involves two key players: the viral reservoir and the immune system. In the typical scenario of chronic infection with an average/large viral reservoir and an impaired immune system, some of the approaches that have been successful showed the ability to target both the viral reservoir and the immune system through gradual (auranofin) or abrupt (chemotherapy/allogeneic transplant) immune system renovation, followed by enhanced immune responses either against conserved viral antigens (auranofin + BSO) or the host’s infected cells (graft versus host disease). In this regard, further studies, fully controlled and with larger number of subjects, will be required to assess the curative potential of the aforementioned strategies. The ultimate goal will be to obtain with scalable drug combinations, the cure that has been induced with more aggressive approaches.

**Table 1:** Summary of the main characteristics of the therapeutic strategies described in this review

	**Notable Results**	**Stage**	**Safety**	**Scalability**
**ART during acute infection**	Long-term post-therapy viral load control in a minority of individuals [16,17,23-30].	Clinical/pre-clinical	High	Low (few patients are detected HIV^+^ at acute infection)
**Viral reactivation with HDACI’s**	Possible disruption of latency [48,49,53-55]. No viral reservoir reduction [49].	Clinical/pre-clinical	Medium	High
**Viral reactivation with cytokines**	IL-7 might disrupt latency but replenishes the viral reservoir [57-59].	Clinical	Medium	Medium/high
**Gene therapy for disruption of *CCR5***	Mixed impact on viral load (depending on the genetic background) [66]. Possible immunologic improvement [67].	Clinical	Medium (long-term effects unknown)	Very low
**Allogeneic stem cell transplant**	Likely sterilizing cures in the second “Berlin Patient” [60,61,63] and in the “Boston Patients” [68,69].	Clinical	Very low	Very low
**Addition of auranofin and BSO to ART**	Long-term post-therapy control in chronically SIVmac251 infected macaques [74,81].	Late pre-clinical	Medium/high (good safety profile for individual drugs in humans)	High
**Therapeutic vaccine with whole virus-pulsed dendritic cells**	Post-therapy viral load control in a subset of macaques [88]. Viral load and viral load set-point reduction in a subset of ART-naïve [89,90] and ART-treated patients [91], respectively	Clinical	High	Medium
**Administration of broadly neutralizing antibody/ies**	Long-term post-therapy control in chronically SHIV(env) infected macaques starting from low viral loads [95]	Late pre-clinical	High	High

In square brackets are the references describing the main results for each strategy.

## Abbreviations

ART: Antiretroviral therapy; STI: Structured treatment interruption; HDACI: Histone deacetylase inhibitor; SIV: Simian immunodeficiency virus; SHIV: Simian/human immunodeficiency virus; BSO: Buthionine sulfoximine; TCM: T central memory; TTM: T transitional memory; TEM: T effector memory.

## Competing interests

The Istituto Superiore di Sanità has requested patent rights on the use of the auranofin/BSO combination for treatment of HIV/AIDS.

## Authors’ contributions

AS and ILS developed the overall theory disclosed in this review, analyzed the literature and drafted the manuscript. ILS drew the original figures. All authors read and approved the final manuscript.

## References

[B1] Margolis DM Eradication therapies for HIV infection: time to begin again AIDS Res Hum Retroviruses 2011 27 4 347 353 doi: 10.1089/aid.2011.0017. Review 10.1089/aid.2011.0017 21314240 PMC3065332

[B2] Trono D Van Lint C Rouzioux C Verdin E Barré-Sinoussi F Chun TW Chomont N HIV persistence and the prospect of long-term drug-free remissions for HIV-infected individuals Science 2010 329 5988 174 180 doi: 10.1126/science.1191047. Review 10.1126/science.1191047 20616270

[B3] Kahn JO Walker BD Acute human immunodeficiency virus type 1 infection N Engl J Med 1998 339 1 33 39 Review 10.1056/NEJM199807023390107 9647878

[B4] Pantaleo G Fauci AS Immunopathogenesis of HIV infection Annu Rev Microbiol 1996 50 825 854 Review 10.1146/annurev.micro.50.1.825 8905100

[B5] Finzi D Hermankova M Pierson T Carruth LM Buck C Chaisson RE Quinn TC Chadwick K Margolick J Brookmeyer R Gallant J Markowitz M Ho DD Richman DD Siliciano RF Identification of a reservoir for HIV-1 in patients on highly active antiretroviral therapy Science 1997 278 5341 1295 1300 10.1126/science.278.5341.1295 9360927

[B6] Finzi D Blankson J Siliciano JD Margolick JB Chadwick K Pierson T Smith K Lisziewicz J Lori F Flexner C Quinn TC Chaisson RE Rosenberg E Walker B Gange S Gallant J Siliciano RF Latent infection of CD4^+^ T cells provides a mechanism for lifelong persistence of HIV-1, even in patients on effective combination therapy Nat Med 1999 5 5 512 517 10.1038/8394 10229227

[B7] Migueles SA Weeks KA Nou E Berkley AM Rood JE Osborne CM Hallahan CW Cogliano-Shutta NA Metcalf JA McLaughlin M Kwan R Mican JM Davey RT Jr Connors M Defective human immunodeficiency virus-specific CD8+ T-cell polyfunctionality, proliferation, and cytotoxicity are not restored by antiretroviral therapy J Virol 2009 83 22 11876 11889 doi: 10.1128/JVI.01153-09 10.1128/JVI.01153-09 19726501 PMC2772718

[B8] Chun TW Engel D Berrey MM Shea T Corey L Fauci AS Early establishment of a pool of latently infected, resting CD4(+) T cells during primary HIV-1 infection Proc Natl Acad Sci U S A 1998 95 15 8869 8873 10.1073/pnas.95.15.8869 9671771 PMC21169

[B9] Siliciano JD Kajdas J Finzi D Quinn TC Chadwick K Margolick JB Kovacs C Gange SJ Siliciano RF Long-term follow-up studies confirm the stability of the latent reservoir for HIV-1 in resting CD4^+^ T cells Nat Med 2003 9 6 727 728 10.1038/nm880 12754504

[B10] Donahue DA Wainberg MA Cellular and molecular mechanisms involved in the establishment of HIV-1 latency Retrovirology 2013 10 11 doi: 10.1186/1742-4690-10-11. Review 10.1186/1742-4690-10-11 23375003 PMC3571915

[B11] Blankson JN Persaud D Siliciano RF The challenge of viral reservoirs in HIV-1 infection Annu Rev Med 2002 53 557 593 Review 10.1146/annurev.med.53.082901.104024 11818490

[B12] Chomont N El-Far M Ancuta P Trautmann L Procopio FA Yassine-Diab B Boucher G Boulassel MR Ghattas G Brenchley JM Schacker TW Hill BJ Douek DC Routy JP Haddad EK Sékaly RP HIV reservoir size and persistence are driven by T cell survival and homeostatic proliferation Nat Med 2009 15 8 893 900 doi: 10.1038/nm.1972 10.1038/nm.1972 19543283 PMC2859814

[B13] Koppensteiner H Brack-Werner R Schindler M Macrophages and their relevance in human immunodeficiency virus type I infection Retrovirology 2012 9 82 doi: 10.1186/1742-4690-9-82 10.1186/1742-4690-9-82 23035819 PMC3484033

[B14] Perelman P Johnson WE Roos C Seuánez HN Horvath JE Moreira MA Kessing B Pontius J Roelke M Rumpler Y Schneider MP Silva A O’Brien SJ Pecon-Slattery J A molecular phylogeny of living primates PLoS Genet 2011 7 3 e1001342 doi: 10.1371/journal.pgen.1001342 10.1371/journal.pgen.1001342 21436896 PMC3060065

[B15] Del Prete GQ Lifson JD Considerations in the development of nonhuman primate models of combination antiretroviral therapy for studies of AIDS virus suppression, residual virus, and curative strategies Curr Opin HIV AIDS 2013 8 4 262 272 doi: 10.1097/COH.0b013e328361cf40 23698559 10.1097/COH.0b013e328361cf40 PMC3939607

[B16] Lisziewicz J Rosenberg E Lieberman J Jessen H Lopalco L Siliciano R Walker B Lori F Control of HIV despite the discontinuation of antiretroviral therapy N Engl J Med 1999 340 21 1683 1684 10.1056/NEJM199905273402114 10348681

[B17] Vila J Nugier F Barguès G Vallet T Peyramond D Hamedi-Sangsari F Seigneurin JM Absence of viral rebound after treatment of HIV-infected patients with didanosine and hydroxycarbamide Lancet 1997 350 9078 635 636 10.1016/S0140-6736(97)24035-3 9288048

[B18] Lori F Lewis MG Xu J Varga G Zinn DE Jr Crabbs C Wagner W Greenhouse J Silvera P Yalley-Ogunro J Tinelli C Lisziewicz J Control of SIV rebound through structured treatment interruptions during early infection Science 2000 290 5496 1591 1593 11090360 10.1126/science.290.5496.1591

[B19] Lori F Lisziewicz J Rationale for the use of hydroxyurea as an anti-human immunodeficiency virus drug Clin Infect Dis 2000 30 Suppl 2 S193 S197 Review 10860905 10.1086/313851

[B20] Longhurst HJ Pinching AJ Drug points: pancreatitis associated with hydroxyurea in combination with didanosine BMJ 2001 322 7278 81 10.1136/bmj.322.7278.81 11154621 PMC26596

[B21] Weissman SB Sinclair GI Green CL Fissell WH Hydroxyurea-induced hepatitis in human immunodeficiency virus-positive patients Clin Infect Dis 1999 29 1 223 224 10.1086/520172 10433603

[B22] Lori F De Forni D Katabira E Baev D Maserati R Calarota SA Cahn P Testori M Rakhmanova A Stevens MR VS411 reduced immune activation and HIV-1 RNA levels in 28 days: randomized proof-of-concept study for antiviral-hyperactivation limiting therapeutics PLoS One 2012 7 10 e47485 doi: 10.1371/journal.pone.0047485 10.1371/journal.pone.0047485 23094055 PMC3477169

[B23] Van Rompay KK Trott KA Jayashankar K Geng Y LaBranche CC Johnson JA Landucci G Lipscomb J Tarara RP Canfield DR Heneine W Forthal DN Montefiori D Abel K Prolonged tenofovir treatment of macaques infected with K65R reverse transcriptase mutants of SIV results in the development of antiviral immune responses that control virus replication after drug withdrawal Retrovirology 2012 9 57 doi: 10.1186/1742-4690-9-57 10.1186/1742-4690-9-57 22805180 PMC3419085

[B24] Rosenberg ES Altfeld M Poon SH Phillips MN Wilkes BM Eldridge RL Robbins GK D’Aquila RT Goulder PJ Walker BD Immune control of HIV-1 after early treatment of acute infection Nature 2000 407 6803 523 526 10.1038/35035103 11029005

[B25] Lampe FC Porter K Kaldor J Law M Kinloch-de Loes S Phillips AN CASCADE Collaboration Effect of transient antiretroviral treatment during acute HIV infection: comparison of the quest trial results with CASCADE natural history study Antivir Ther 2007 12 2 189 193 17503661

[B26] Volberding P Demeter L Bosch RJ Aga E Pettinelli C Hirsch M Vogler M Martinez A Little S Connick E ACTG 371 Team Antiretroviral therapy in acute and recent HIV infection: a prospective multicenter stratified trial of intentionally interrupted treatment AIDS 2009 23 15 1987 1995 doi: 10.1097/QAD.0b013e32832eb285 10.1097/QAD.0b013e32832eb285 19696651 PMC2888600

[B27] Hocqueloux L Prazuck T Avettand-Fenoel V Lafeuillade A Cardon B Viard JP Rouzioux C Long-term immunovirologic control following antiretroviral therapy interruption in patients treated at the time of primary HIV-1 infection AIDS 2010 24 10 1598 1601 10.1097/QAD.0b013e32833b61ba 20549847

[B28] Lodi S Meyer L Kelleher AD Rosinska M Ghosn J Sannes M Porter K Immunovirologic control 24 months after interruption of antiretroviral therapy initiated close to HIV seroconversion Arch Intern Med 2012 172 16 1252 1255 doi: 10.1001/archinternmed.2012.2719 10.1001/archinternmed.2012.2719 22826124

[B29] Sáez-Cirión A Bacchus C Hocqueloux L Avettand-Fenoel V Girault I Lecuroux C Potard V Versmisse P Melard A Prazuck T Descours B Guergnon J Viard JP Boufassa F Lambotte O Goujard C Meyer L Costagliola D Venet A Pancino G Autran B Rouzioux C Post-treatment HIV-1 controllers with a long-term virological remission after the interruption of early initiated antiretroviral therapy ANRS VISCONTI Study PLoS Pathog 2013 9 3 e1003211 doi: 10.1371/journal.ppat.1003211 10.1371/journal.ppat.1003211 23516360 PMC3597518

[B30] Persaud D Gay H Ziemniak C Chen YH Piatak M Jr Chun TW Strain M Richman D Luzuriaga K Absence of detectable HIV-1 viremia after treatment cessation in an infant N Engl J Med 2013 369 19 1828 1835 doi: 10.1056/NEJMoa1302976 10.1056/NEJMoa1302976 24152233 PMC3954754

[B31] Desquilbet L Goujard C Rouzioux C Sinet M Deveau C Chaix ML Séréni D Boufassa F Delfraissy JF Meyer L PRIMO and SEROCO Study Groups Does transient HAART during primary HIV-1 infection lower the virological set-point? AIDS 2004 18 18 2361 2369 15622312

[B32] Streeck H Jessen H Alter G Teigen N Waring MT Jessen A Stahmer I van Lunzen J Lichterfeld M Gao X Allen TM Carrington M Walker BD Rockstroh JK Altfeld M Immunological and virological impact of highly active antiretroviral therapy initiated during acute HIV-1 infection J Infect Dis 2006 194 6 734 739 10.1086/503811 16941338

[B33] Pantazis N Touloumi G Vanhems P Gill J Bucher HC Porter K CASCADE Collaboration The effect of antiretroviral treatment of different durations in primary HIV infection AIDS 2008 22 18 2441 2450 doi: 10.1097/QAD.0b013e328319ea4e 10.1097/QAD.0b013e328319ea4e 19005267

[B34] Oxenius A Price DA Easterbrook PJ O’Callaghan CA Kelleher AD Whelan JA Sontag G Sewell AK Phillips RE Early highly active antiretroviral therapy for acute HIV-1 infection preserves immune function of CD8+ and CD4+ T lymphocytes Proc Natl Acad Sci U S A 2000 97 7 3382 3387 10.1073/pnas.97.7.3382 10737796 PMC16248

[B35] Moir S Buckner CM Ho J Wang W Chen J Waldner AJ Posada JG Kardava L O’Shea MA Kottilil S Chun TW Proschan MA Fauci AS B cells in early and chronic HIV infection: evidence for preservation of immune function associated with early initiation of antiretroviral therapy Blood 2010 116 25 5571 5579 doi: 10.1182/blood-2010-05-285528 10.1182/blood-2010-05-285528 20837780 PMC3031405

[B36] Ngo-Giang-Huong N Deveau C Da Silva I Pellegrin I Venet A Harzic M Sinet M Delfraissy JF Meyer L Goujard C Rouzioux C French PRIMO Cohort Study Group Proviral HIV-1 DNA in subjects followed since primary HIV-1 infection who suppress plasma viral load after one year of highly active antiretroviral therapy AIDS 2001 15 6 665 673 10.1097/00002030-200104130-00001 11371680

[B37] Archin NM Vaidya NK Kuruc JD Liberty AL Wiegand A Kearney MF Cohen MS Coffin JM Bosch RJ Gay CL Eron JJ Margolis DM Perelson AS Immediate antiviral therapy appears to restrict resting CD4^+^ cell HIV-1 infection without accelerating the decay of latent infection Proc Natl Acad Sci U S A 2012 109 24 9523 9528 doi: 10.1073/pnas.1120248109 10.1073/pnas.1120248109 22645358 PMC3386138

[B38] Jain V Hartogensis W Bacchetti P Hunt PW Hatano H Sinclair E Epling L Lee TH Busch MP McCune JM Pilcher CD Hecht FM Deeks SG Antiretroviral therapy initiated within Six months of HIV infection is associated with lower T-cell activation and smaller HIV reservoir size J Infect Dis 2013 208 8 1202 1211 doi: 10.1093/infdis/jit311 10.1093/infdis/jit311 23852127 PMC3778965

[B39] Chun TW Justement JS Murray D Hallahan CW Maenza J Collier AC Sheth PM Kaul R Ostrowski M Moir S Kovacs C Fauci AS Rebound of plasma viremia following cessation of antiretroviral therapy despite profoundly low levels of HIV reservoir: implications for eradication AIDS 2010 24 18 2803 2808 doi: 10.1097/QAD.0b013e328340a239 10.1097/QAD.0b013e328340a239 20962613 PMC3154092

[B40] Dybul M Nies-Kraske E Daucher M Hertogs K Hallahan CW Csako G Yoder C Ehler L Sklar PA Belson M Hidalgo B Metcalf JA Davey RT Rock Kress DM Powers A Fauci AS Long-cycle structured intermittent versus continuous highly active antiretroviral therapy for the treatment of chronic infection with human immunodeficiency virus: effects on drug toxicity and on immunologic and virologic parameters J Infect Dis 2003 188 3 388 396 10.1086/376535 12870120

[B41] Alexander TH Ortiz GM Wellons MF Allen A Grace EJ 2nd Schweighardt B Brancato J Sandberg JK Furlan SN Miralles GD Nixon DF Bartlett JA Changes in CD4^+^ T-cell differentiation phenotype during structured treatment interruption in patients with chronic HIV-1 infection J Acquir Immune Defic Syndr 2003 34 5 475 481 10.1097/00126334-200312150-00005 14657757

[B42] Papasavvas E Kostman JR Mounzer K Grant RM Gross R Gallo C Azzoni L Foulkes A Thiel B Pistilli M Mackiewicz A Shull J Montaner LJ Randomized, controlled trial of therapy interruption in chronic HIV-1 infection PLoS Med 2004 1 3 e64 10.1371/journal.pmed.0010064 15630469 PMC539050

[B43] Pai NP Tulsky JP Lawrence J Colford JM Jr Reingold AL Structured treatment interruptions (STI) in chronic suppressed HIV infection in adults Cochrane Database Syst Rev 2005 2005 4 CD005482 Review 16235406 10.1002/14651858.CD005482

[B44] Hamer DH Can HIV, be cured? Mechanisms of HIV persistence and strategies to combat it Curr HIV Res 2004 2 2 99 111 Review 10.2174/1570162043484915 15078175

[B45] Van Lint C Bouchat S Marcello A HIV-1 transcription and latency: an update Retrovirology 2013 10 67 doi: 10.1186/1742-4690-10-67. Review 10.1186/1742-4690-10-67 23803414 PMC3699421

[B46] Sgarbanti M Battistini A Therapeutics for HIV-1 reactivation from latency Curr Opin Virol 2013 3 4 394 401 doi: 10.1016/j.coviro.2013.06.001. Review 10.1016/j.coviro.2013.06.001 23810462

[B47] Wightman F Ellenberg P Churchill M Lewin SR HDAC inhibitors in HIV Immunol Cell Biol 2012 90 1 47 54 doi: 10.1038/icb.2011.95. Review 10.1038/icb.2011.95 22083528

[B48] Archin NM Liberty AL Kashuba AD Choudhary SK Kuruc JD Crooks AM Parker DC Anderson EM Kearney MF Strain MC Richman DD Hudgens MG Bosch RJ Coffin JM Eron JJ Hazuda DJ Margolis DM Administration of vorinostat disrupts HIV-1 latency in patients on antiretroviral therapy Nature 2012 487 7408 482 485 doi: 10.1038/nature11286. Erratum in: Nature. 2012; **489**(7416):460 10.1038/nature11286 22837004 PMC3704185

[B49] Elliott J Solomon A Wightman F Smith M Palmer S Prince M Watson J Hoy J McMahon J Lewin SR The safety and effect of multiple doses of vorinostat on HIV transcription in HIV + patients receiving cART20th Conference on Retroviruses and Opportunistic Infections (CROI) 2013 Atlanta, USA: Oral late breaker abstract LB50

[B50] Monforte AD Svicher V Nozza S Lazzarin A Marchetti G Perno CF Highlights on HIV eradication in 2013 AIDS 2013 [Epub ahead of print] 10.1097/01.aids.0000433241.78739.79 23939240

[B51] Lifson J Del Prete G Kiser R Trubey CM Smedley J Coalter V Oswald K Shoemaker R Fast R Li Y Lara A Wiles A Wiles R Macallister R Sanchez R Wai J Tan C Keele B Estes J Piatak M Jr Hazuda DJ Evaluation of treatment with the histone deacetylase inhibitor vorinostat (suberoylanilide hydroxamic acid; SAHA) in antiretroviral drug treated, SIVmac239-infected Rhesus Macaques Towards a Cure: IAS pre-conference symposium 2012 Washington D.C.: Oral presentation

[B52] Lewis MG DaFonseca S Chomont N Palamara AT Tardugno M Mai A Collins M Wagner WL Yalley-Ogunro J Greenhouse J Chirullo B Norelli S Garaci E Savarino A Gold drug auranofin restricts the viral reservoir in the monkey AIDS model and induces containment of viral load following ART suspension AIDS 2011 25 11 1347 1356 doi: 10.1097/QAD.0b013e328347bd77 10.1097/QAD.0b013e328347bd77 21505294

[B53] Savarino A Mai A Norelli S El Daker S Valente S Rotili D Altucci L Palamara AT Garaci E “Shock and kill” effects of class I-selective histone deacetylase inhibitors in combination with the glutathione synthesis inhibitor buthionine sulfoximine in cell line models for HIV-1 quiescence Retrovirology 2009 6 52 doi: 10.1186/1742-4690-6-52 10.1186/1742-4690-6-52 19486542 PMC2697151

[B54] Rasmussen TA Schmeltz Søgaard O Brinkmann C Wightman F Lewin SR Melchjorsen J Dinarello C Ostergaard L Tolstrup M Comparison of HDAC inhibitors in clinical development: effect on HIV production in latently infected cells and T-cell activation Hum Vaccin Immunother 2013 9 5 993 1001 doi: 10.4161/hv.23800 10.4161/hv.23800 23370291 PMC3899169

[B55] Shan L Xing S Yang HC Zhang H Margolick JB Siliciano RF Unique characteristics of histone deacetylase inhibitors in reactivation of latent HIV-1 in Bcl-2-transduced primary resting CD4+ T cells J Antimicrob Chemother 2013 [Epub ahead of print] 10.1093/jac/dkt338 PMC3861332 23999005

[B56] Vandergeeten C Fromentin R Chomont N The role of cytokines in the establishment, persistence and eradication of the HIV reservoir Cytokine Growth Factor Rev 2012 23 4–5 143 149 doi: 10.1016/j.cytogfr.2012.05.001. Review 22743037 10.1016/j.cytogfr.2012.05.001 PMC3767481

[B57] Imamichi H Degray G Asmuth DM Fischl MA Landay AL Lederman MM Sereti I HIV-1 viruses detected during episodic blips following interleukin-7 administration are similar to the viruses present before and after interleukin-7 therapy AIDS 2011 25 2 159 164 doi: 10.1097/QAD.0b013e328340a270 10.1097/QAD.0b013e328340a270 21124203 PMC3074174

[B58] Vandergeeten C Fromentin R DaFonseca S Lawani MB Sereti I Lederman MM Ramgopal M Routy JP Sékaly RP Chomont N Interleukin-7 promotes HIV persistence during antiretroviral therapy Blood 2013 121 21 4321 4329 doi: 10.1182/blood-2012-11-465625 10.1182/blood-2012-11-465625 23589672 PMC3663425

[B59] Bosque A Famiglietti M Weyrich AS Goulston C Planelles V Homeostatic proliferation fails to efficiently reactivate HIV-1 latently infected central memory CD4^+^ T cells PLoS Pathog 2011 7 10 e1002288 doi: 10.1371/journal.ppat.1002288 10.1371/journal.ppat.1002288 21998586 PMC3188522

[B60] Hütter G Nowak D Mossner M Ganepola S Müssig A Allers K Schneider T Hofmann J Kücherer C Blau O Blau IW Hofmann WK Thiel E Long-term control of HIV by CCR5 Delta32/Delta32 stem-cell transplantation N Engl J Med 2009 360 7 692 698 doi: 10.1056/NEJMoa0802905 10.1056/NEJMoa0802905 19213682

[B61] Allers K Hütter G Hofmann J Loddenkemper C Rieger K Thiel E Schneider T Evidence for the cure of HIV infection by CCR5Δ32/Δ32 stem cell transplantation Blood 2011 117 10 2791 2799 doi: 10.1182/blood-2010-09-309591 10.1182/blood-2010-09-309591 21148083

[B62] Dean M Carrington M Winkler C Huttley GA Smith MW Allikmets R Goedert JJ Buchbinder SP Vittinghoff E Gomperts E Donfield S Vlahov D Kaslow R Saah A Rinaldo C Detels R O'Brien SJ Genetic restriction of HIV-1 infection and progression to AIDS by a deletion allele of the CKR5 structural gene. Hemophilia Growth and Development Study, Multicenter AIDS Cohort Study, Multicenter Hemophilia Cohort Study, San Francisco City Cohort, ALIVE Study Science 1996 273 5283 1856 62 Erratum in: Science 1996; 274(5290):1069 10.1126/science.273.5283.1856 8791590

[B63] Yukl SA Boritz E Busch M Bentsen C Chun TW Douek D Eisele E Haase A Ho YC Hütter G Justement JS Keating S Lee TH Li P Murray D Palmer S Pilcher C Pillai S Price RW Rothenberger M Schacker T Siliciano J Siliciano R Sinclair E Strain M Wong J Richman D Deeks SG Challenges in detecting HIV persistence during potentially curative interventions: a study of the Berlin patient PLoS Pathog 2013 9 5 e1003347 doi: 10.1371/journal.ppat.1003347 10.1371/journal.ppat.1003347 23671416 PMC3649997

[B64] Hütter G Zaia JA Allogeneic haematopoietic stem cell transplantation in patients with human immunodeficiency virus: the experiences of more than 25 years Clin Exp Immunol 2011 163 3 284 295 doi: 10.1111/j.1365-2249.2010.04312.x. Review 10.1111/j.1365-2249.2010.04312.x 21303358 PMC3048611

[B65] Huzicka I Could bone marrow transplantation cure AIDS?: review Med Hypotheses 1999 52 3 247 257 10.1054/mehy.1997.0638 10362285

[B66] Ando D Lalezari J Blick G Rodriquez J Hsu R Hawkins T Parks D Zeidan J Sekaly RP Deeks S Functional control of viremia in CCR5-Δ32 heterozygous (Δ32HZ) HIV^+^ subjects following adoptive transfer of zinc finger nuclease CCR5 modified autologous CD4 T-cells (SB-728-T) 53rd Interscience Conference on Antimicrobial Agents and Chemotherapy (ICAAC) 2013 Denver, USA

[B67] Lee GK Zeidan J Lalezari J Mitsuyasu R Wang S Giedlin M Nichol G Tang W Ando D Sekaly RP Long Term CD4 Reconstitution in HIV Subjects Receiving ZFN CCR5 Modified CD4 T-Cells (SB-728-T) May Be Attributed to the Sustained Durability of the Central Memory T-Cell Subset20th Conference on Retroviruses and Opportunistic Infections (CROI) 2013 Atlanta, USA Abstract #126

[B68] Henrich TJ Hu Z Li JZ Sciaranghella G Busch MP Keating SM Gallien S Lin NH Giguel FF Lavoie L Ho VT Armand P Soiffer RJ Sagar M Lacasce AS Kuritzkes DR Long-term reduction in peripheral blood HIV type 1 reservoirs following reduced-intensity conditioning allogeneic stem cell transplantation J Infect Dis 2013 207 11 1694 1702 doi: 10.1093/infdis/jit086 10.1093/infdis/jit086 23460751 PMC3636784

[B69] Henrich T Hanhauser E Sirignano M Davis B Lee TH Keating S Busch M Marty F LaCasce A Armand P Soiffer R Altfeld M Kuritzkes D In depth investigation of peripheral and gut HIV-1 reservoirs, HIV-specific cellular immunity, and host microchimerism following allogeneic hematopoetic stem cell transplantation7th IAS Conference on HIV Pathogenesis, Treatment and Prevention 2013 Kuala Lumpur, Malaysia Abstract WELBA05

[B70] Stewart GJ Ashton LJ Biti RA Ffrench RA Bennetts BH Newcombe NR Benson EM Carr A Cooper DA Kaldor JM Increased frequency of CCR-5 delta 32 heterozygotes among long-term non-progressors with HIV-1 infection. The Australian long-term Non-progressor study group AIDS 1997 11 15 1833 1838 10.1097/00002030-199715000-00007 9412701

[B71] Paiardini M Cervasi B Reyes-Aviles E Micci L Ortiz AM Chahroudi A Vinton C Gordon SN Bosinger SE Francella N Hallberg PL Cramer E Schlub T Chan ML Riddick NE Collman RG Apetrei C Pandrea I Else J Munch J Kirchhoff F Davenport MP Brenchley JM Silvestri G Low levels of SIV infection in sooty mangabey central memory CD4^+^ T cells are associated with limited CCR5 expression Nat Med 2011 17 7 830 836 doi: 10.1038/nm.2395 10.1038/nm.2395 21706028 PMC3253129

[B72] Jenq RR van den Brink MR Allogeneic haematopoietic stem cell transplantation: individualized stem cell and immune therapy of cancer Nat Rev Cancer 2010 10 3 213 221 doi: 10.1038/nrc2825. Review. Erratum in: *Nat Rev Cancer.* 2010;**10**(3) 10.1038/nrc2804 20168320

[B73] Suarez-Almazor ME Spooner CH Belseck E Shea B Auranofin versus placebo in rheumatoid arthritis Cochrane Database Syst Rev 2000 2000 2 CD002048 Review 10796461 10.1002/14651858.CD002048 PMC8436883

[B74] Shytaj IL Norelli S Chirullo B Della Corte A Collins M Yalley-Ogunro J Greenhouse J Iraci N Acosta EP Barreca ML Lewis MG Savarino A A highly intensified ART regimen induces long-term viral suppression and restriction of the viral reservoir in a simian AIDS model PLoS Pathog 2012 8 6 e1002774 doi: 10.1371/journal.ppat.1002774 10.1371/journal.ppat.1002774 22737073 PMC3380955

[B75] Vanham G Van Gulck E Can immunotherapy be useful as a “functional cure” for infection with Human Immunodeficiency Virus-1? Retrovirology 2012 9 72 doi: 10.1186/1742-4690-9-72. Review 10.1186/1742-4690-9-72 22958464 PMC3472319

[B76] Betts MR Nason MC West SM De Rosa SC Migueles SA Abraham J Lederman MM Benito JM Goepfert PA Connors M Roederer M Koup RA HIV nonprogressors preferentially maintain highly functional HIV-specific CD8^+^ T cells Blood 2006 107 12 4781 4789 10.1182/blood-2005-12-4818 16467198 PMC1895811

[B77] Almeida JR Price DA Papagno L Arkoub ZA Sauce D Bornstein E Asher TE Samri A Schnuriger A Theodorou I Costagliola D Rouzioux C Agut H Marcelin AG Douek D Autran B Appay V Superior control of HIV-1 replication by CD8^+^ T cells is reflected by their avidity, polyfunctionality, and clonal turnover J Exp Med 2007 204 10 2473 2485 10.1084/jem.20070784 17893201 PMC2118466

[B78] Hersperger AR Pereyra F Nason M Demers K Sheth P Shin LY Kovacs CM Rodriguez B Sieg SF Teixeira-Johnson L Gudonis D Goepfert PA Lederman MM Frank I Makedonas G Kaul R Walker BD Betts MR Perforin expression directly ex vivo by HIV-specific CD8 T-cells is a correlate of HIV elite control PLoS Pathog 2010 6 5 e1000917 doi: 10.1371/journal.ppat.1000917 10.1371/journal.ppat.1000917 20523897 PMC2877741

[B79] Buckheit RW 3rd Siliciano RF Blankson JN Primary CD8^+^ T cells from elite suppressors effectively eliminate non-productively HIV-1 infected resting and activated CD4^+^ T cells Retrovirology 2013 10 68 doi: 10.1186/1742-4690-10-68 10.1186/1742-4690-10-68 23816179 PMC3702406

[B80] Bailey HH Ripple G Tutsch KD Arzoomanian RZ Alberti D Feierabend C Mahvi D Schink J Pomplun M Mulcahy RT Wilding G Phase I study of continuous-infusion L-S, R-buthionine sulfoximine with intravenous melphalan J Natl Cancer Inst 1997 89 23 1789 1796 10.1093/jnci/89.23.1789 9392620

[B81] Shytaj IL Chirullo B Wagner W Ferrari MG Sgarbanti R Corte AD LaBranche C Lopalco L Palamara AT Montefiori D Lewis MG Garaci E Savarino A Investigational treatment suspension and enhanced cell-mediated immunity at rebound followed by drug-free remission of simian AIDS Retrovirology 2013 10 71 doi: 10.1186/1742-4690-10-71 10.1186/1742-4690-10-71 23866829 PMC3748827

[B82] Stephenson KE Li H Walker BD Michael NL Barouch DH Gag-specific cellular immunity determines in vitro viral inhibition and in vivo virologic control following simian immunodeficiency virus challenges of vaccinated rhesus monkeys J Virol 2012 86 18 9583 9589 doi: 10.1128/JVI.00996-12 10.1128/JVI.00996-12 22761379 PMC3446565

[B83] Edwards BH Bansal A Sabbaj S Bakari J Mulligan MJ Goepfert PA Magnitude of functional CD8^+^ T-cell responses to the gag protein of human immunodeficiency virus type 1 correlates inversely with viral load in plasma J Virol 2002 76 5 2298 2305 10.1128/jvi.76.5.2298-2305.2002 11836408 PMC135950

[B84] Zuñiga R Lucchetti A Galvan P Sanchez S Sanchez C Hernandez A Sanchez H Frahm N Linde CH Hewitt HS Hildebrand W Altfeld M Allen TM Walker BD Korber BT Leitner T Sanchez J Brander C Relative dominance of Gag p24-specific cytotoxic T lymphocytes is associated with human immunodeficiency virus control J Virol 2006 80 6 3122 3125 10.1128/JVI.80.6.3122-3125.2006 16501126 PMC1395458

[B85] Kiepiela P Ngumbela K Thobakgale C Ramduth D Honeyborne I Moodley E Reddy S de Pierres C Mncube Z Mkhwanazi N Bishop K van der Stok M Nair K Khan N Crawford H Payne R Leslie A Prado J Prendergast A Frater J McCarthy N Brander C Learn GH Nickle D Rousseau C Coovadia H Mullins JI Heckerman D Walker BD Goulder P CD8^+^ T-cell responses to different HIV proteins have discordant associations with viral load Nat Med 2007 13 1 46 53 10.1038/nm1520 17173051

[B86] Julg B Williams KL Reddy S Bishop K Qi Y Carrington M Goulder PJ Ndung’u T Walker BD Enhanced anti-HIV functional activity associated with Gag-specific CD8 T-cell responses J Virol 2010 84 11 5540 5549 doi: 10.1128/JVI.02031-09 10.1128/JVI.02031-09 20335261 PMC2876607

[B87] Pace MJ Graf EH Agosto LM Mexas AM Male F Brady T Bushman FD O’Doherty U Directly infected resting CD4^+^T cells can produce HIV Gag without spreading infection in a model of HIV latency PLoS Pathog 2012 8 7 e1002818 doi: 10.1371/journal.ppat.1002818 10.1371/journal.ppat.1002818 22911005 PMC3406090

[B88] Lu W Wu X Lu Y Guo W Andrieu JM Therapeutic dendritic-cell vaccine for simian AIDS Nat Med 2003 9 1 27 32 12496959 10.1038/nm806

[B89] Lu W Arraes LC Ferreira WT Andrieu JM Therapeutic dendritic-cell vaccine for chronic HIV-1 infection Nat Med 2004 10 12 1359 1365 10.1038/nm1147 15568033

[B90] García F Climent N Assoumou L Gil C González N Alcamí J León A Romeu J Dalmau J Martínez-Picado J Lifson J Autran B Costagliola D Clotet B Gatell JM Plana M Gallart T DCV2/MANON07- AIDS Vaccine Research Objective Study Group A therapeutic dendritic cell-based vaccine for HIV-1 infection J Infect Dis 2011 203 4 473 478 10.1093/infdis/jiq077 21233310 PMC3071229

[B91] García F Climent N Guardo AC Gil C León A Autran B Lifson JD Martínez-Picado J Dalmau J Clotet B Gatell JM Plana M Gallart T DCV2/MANON07-ORVACS Study Group A dendritic cell-based vaccine elicits T cell responses associated with control of HIV-1 replication Sci Transl Med 2013 5 166 166ra2 doi: 10.1126/scitranslmed.3004682 23283367 10.1126/scitranslmed.3004682

[B92] Hansen SG MP Jr Ventura AB Hughes CM Gilbride RM Ford JC Oswald K Shoemaker R Li Y Lewis MS Gilliam AN Xu G Whizin N Burwitz BJ Planer SL Turner JM Legasse AW Axthelm MK Nelson JA Früh K Sacha JB Estes JD Keele BF Edlefsen PT Lifson JD Picker LJ Immune clearance of highly pathogenic SIV infection Nature 2013 502 7469 100 104 doi: 10.1038/nature12519 10.1038/nature12519 24025770 PMC3849456

[B93] Hansen SG Ford JC Lewis MS Ventura AB Hughes CM Coyne-Johnson L Whizin N Oswald K Shoemaker R Swanson T Legasse AW Chiuchiolo MJ Parks CL Axthelm MK Nelson JA Jarvis MA Piatak M Jr Lifson JD Picker LJ Profound early control of highly pathogenic SIV by an effector memory T-cell vaccine Nature 2011 473 7348 523 527 doi: 10.1038/nature10003 10.1038/nature10003 21562493 PMC3102768

[B94] Vagenas P Aravantinou M Williams VG Jasny E Piatak M Jr Lifson JD Salazar AM Blanchard JL Gettie A Robbiani M A tonsillar PolyICLC/AT-2 SIV therapeutic vaccine maintains low viremia following antiretroviral therapy cessation PLoS One 2010 5 9 e12891 doi: 10.1371/journal.pone.0012891 10.1371/journal.pone.0012891 20877632 PMC2943484

[B95] Barouch DH Whitney JB Moldt B Klein F Oliveira TY Liu J Stephenson KE Chang HW Shekhar K Gupta S Nkolola JP Seaman MS Smith KM Borducchi EN Cabral C Smith JY Blackmore S Sanisetty S Perry JR Beck M Lewis MG Rinaldi W Chakraborty AK Poignard P Nussenzweig MC Burton DR Therapeutic efficacy of potent neutralizing HIV-1-specific monoclonal antibodies in SHIV-infected rhesus monkeys Nature 2013 doi: 10.1038/nature12744. [Epub ahead of print] 10.1038/nature12744 PMC4017780 24172905

[B96] Villinger F Brice GT Mayne AE Bostik P Mori K June CH Ansari AA Adoptive transfer of simian immunodeficiency virus (SIV) naïve autologous CD4(+) cells to macaques chronically infected with SIV is sufficient to induce long-term nonprogressor status Blood 2002 99 2 590 599 10.1182/blood.V99.2.590 11781243

[B97] Cillo AR Krishnan A Mitsuyasu RT McMahon DK Li S Rossi JJ Zaia JA Mellors JW Plasma viremia and cellular HIV-1 DNA persist despite autologous hematopoietic stem cell transplantation for HIV-related lymphoma J Acquir Immune Defic Syndr 2013 63 4 438 441 doi: 10.1097/QAI.0b013e31828e6163 10.1097/QAI.0b013e31828e6163 23493152 PMC3699958

